# Survival of the fastest? A descriptive analysis of severely injured trauma patients primarily admitted or secondarily transferred to major trauma centers in a Danish inclusive trauma system

**DOI:** 10.1186/s13049-024-01265-3

**Published:** 2024-09-14

**Authors:** Thea Palsgaard Møller, Josefine Tangen Jensen, Roar Borregaard Medici, Søren Steemann Rudolph, Lars Bredevang Andersen, Jakob Roed, Stig Nikolaj Fasmer Blomberg, Helle Collatz Christensen, Mark Edwards

**Affiliations:** 1 Prehospital Center, Region Zealand, Ringstedgade 61, 13th Floor, 4700 Næstved, Denmark; 2 Department of Anesthesiology and Intensive Care Medicine, Holbæk Hospital, Region Zealand, Holbæk, Denmark; 3 Institute of Clinical Medicine, University of Copenhagen, Copenhagen, Denmark; 4 Department of Anaesthesia and Trauma Center, Centre of Head and Orthopaedics 6011, Copenhagen University Hospital Rigshospitalet, Copenhagen, Denmark; 5 Department of Anesthesiology and Intensive Care Medicine, Zealand University Hospital Roskilde, Region Zealand, Roskilde, Denmark; 6 Queen Mary University of London, London, UK

## Abstract

**Background:**

Trauma systems are crucial for enhancing survival and quality of life for trauma patients. Understanding trauma triage and patient outcomes is essential for optimizing resource allocation and trauma care.

**Aims:**

The aim was to explore prehospital trauma triage in Region Zealand, Denmark. Specifically, characteristics for patients who were either primarily admitted or secondarily transferred to major trauma centers were described.

**Methods:**

A retrospective descriptive study of severely injured trauma patients was conducted from January 2017 to December 2021.

**Results:**

The study comprised 744 patients including 55.6% primary and 44.4% secondary patients. Overall, men accounted for 70.2% of patients, and 66.1% were aged 18–65 years. The secondary patients included more women—34.2% versus 26.3% and a higher proportion of Injury Severity Score of ≥ 15—59.6% versus 47.8%, compared to primary patients. 30-day survival was higher for secondary patients—92.7% versus 87%. Medical dispatchers assessed urgency as Emergency level A for 98.1% of primary patients and 86.3% for secondary patients. Physician-staffed prehospital units attended primary patients first more frequently—17.1% versus 3.5%. Response times were similar, but time at scene was longer for primary patients whereas time from injury to arrival at a major trauma center was longer for secondary patients.

**Conclusions:**

Secondary trauma patients had higher Injury Severity Scores and better survival rates. They were considered less urgent by medical dispatchers and less frequently assessed by physician-staffed units. Prospective quality data are needed for further investigation of optimal triage and continuous quality improvement in trauma care.

## Background

Trauma is the leading cause of death in the Western world among people under 45 years of age and a major public health concern [1, 2]. Trauma systems, which are comprehensive infrastructures aiming at providing optimal care, have been shown to decrease injury mortality and morbidity for trauma patients in general and are important in terms of securing high quality in the regional, multidisciplinary response to injury[3].

Within trauma systems, prehospital triage to the most appropriate facility is a main concern. Accurate triage is crucial to balancing the delivery of cost-effective and appropriate level of care. European Guidelines highlight the importance of bringing severely injured patients directly to a major trauma center (MTC) and minimizing the elapse between injury and bleeding control [4]. Ideally, trauma patients are transported to a hospital capable of addressing their exact injuries without risking long transport times, or secondary transfer, while avoiding unnecessary activation of costly trauma teams [3]. Undertriage is associated with increased mortality [5, 6], while overtriage leads to significant resource overuse [7–9]. The American College of Surgeons Committee of Trauma suggests targets of 5% for undertriage and 35% for overtriage in their 2021 guidelines [10]. A 2018 trauma study of severely injured adults with an Injury Severity Score (ISS) of 16 or greater found 30.6% overtriage and 21.6% undertriage rates, indicating a large proportion of patients prone to preventable adverse outcomes [11]. Other studies show that undertriage is particularly frequent in older patients [12] and children [13].

Despite major improvements in trauma systems during the past decades, prehospital trauma triage remains challenging. Limited research exists on the optimal triage of trauma patients, but improving our understanding would enhance our foundation for optimizing resource allocation and care within the system. Notably, a 2009 Danish study revealed that severely injured trauma patients with ISS more than 15 had higher mortality rates when treated at local hospitals compared to those secondarily transferred to and treated at a major trauma center [14]. Additionally, a recent Danish study found that trauma patients transferred from trauma units to MTCs had a median time of 255 min from injury to MTC arrival [15]. While this delay might appear significant within the Danish context, its impact on patient outcomes remains uncertain. Further investigation is needed to understand potential differences in outcomes between patients primarily admitted to MTCs versus those transferred secondarily, including analyzing the distinct characteristics of these two patient groups.

The overall objective of the current study was to explore prehospital trauma triage in the inclusive trauma system in Region Zealand, Denmark. This was done with an exploratory and hypothesis-generating purpose. The primary aim was to describe patient characteristics and prehospital characteristics for severely injured trauma patients who were either primarily admitted (“primary patients”) or secondarily transferred (“secondary patients”) to an MTC in adjacent regions (Copenhagen or Odense).

## Methods

We performed a retrospective descriptive study of severely injured trauma patients from Region Zealand who were either primarily admitted or secondarily transferred to the MTC in adjacent regions (Copenhagen or Odense) in a 5-year study period from 1 January 2017 to 31 December 2021. A 5-year period was chosen to ensure a representative sample of the trauma population and at the same time to consider any effect of COVID-19, which may have affected the flow of trauma patients during the period. The results are presented in accordance with the Strengthening the Reporting of Observational Studies in Epidemiology (STROBE) guidelines [16].

### Study site and setting

In Denmark, all citizens have tax-funded and open access to health care via a single emergency phone number. Medical calls are directed to one of five regional emergency medical dispatch centres. Medical dispatchers (specially trained nurses and paramedics) at each centre use a criteria-based dispatch tool [17] to prioritise the incoming calls and, if required, provide pre-arrival instructions to callers until arrival of emergency services. The dispatch tool contains a mandatory assessment and registration of the contact cause and degree of urgency for the emergency call. Based on this assessment, the system suggests a response type and accompanying competence to be dispatched to the individual incident [18]. At the dispatch centre, a physician on-call provides backup for the medical dispatchers and the ambulance personnel in the region whenever needed. All five Danish regions have a two-tiered response system with physician- and paramedic-staffed mobile critical care units (MCCU) in addition to ambulances staffed with paramedics and emergency medical technicians. However, the dispatch criteria of the MCCU’s varies between regions. In Region Zealand, an administrative region of 7273 km^2^, two MCCU’s are available for interhospital transport of severely ill or injured patients and call-outs for primary missions. A national Helicopter emergency medical service (HEMS) supports the trauma system.

Region Zealand is the only region of the five Danish regions that does not have an MTC with multidisciplinary advanced trauma care including competencies within neurosurgery, cardio-thoracic surgery, and paediatric surgery. In the region there are four hospitals with trauma units capable of managing minor trauma or, in case of major trauma, resuscitating and stabilizing patients prior to transport to an MTC. In case of trauma, the ambulance personnel will triage the patients according to predefined triage criteria (see Appendix for prehospital trauma triage in Region Zealand to Trauma Units and/or MTC) either to a local trauma unit or to an MTC located outside the region (see Fig. 2 for a map of Region Zealand and neighbouring regions). The choice of which MTC to drive to depends on the location of the trauma and the distance to the MTC. Severely injured patients admitted primarily to a trauma unit may need secondary transfer to an MTC after initial assessment and stabilization [19].

### Data collection and management

Severely injured patients were identified in the Danish Trauma Registry (DTR) from which data were obtained [20]. This registry was only recently established (2014) and at this point only contains data from MTCs. A future extension is planned to comprise data from all Danish trauma units. The registry aims to monitor and improve the quality of trauma treatment in Danish hospitals to increase survival and minimize lasting consequences for trauma patients. Variables such as patient age, gender, injury mechanism, Injury Severity Score (ISS) [21], Charlson Comorbidity Index [22], and mortality were obtained from DTR.

Prehospital data were obtained from the administrative database of the emergency medical services in Region Zealand [23]. The personal identification number which is unique for all Danish citizens [24] and the date and time of the trauma call at the MTC were used to link data from the trauma dataset with prehospital data. Trauma patients from Region Zealand were included if they were transported by a pre-hospital resource from the region within 2 days before arriving at the MTC. Data was stored in a legal and secure research database, from where data management and statistical analysis were performed.

### Inclusion criteria

The study encompassed trauma patients across all age groups who sustained injuries within the Zealand Region regardless of their residency. This included individuals who were either initially transported and admitted or subsequently transferred to an MTC outside the region. Secondary patients, in this context, were exclusively those transferred from one of the four university hospitals in the Zealand Region, where primary admission occurred. The study period was 5 years from January 2017 to December 2021.

### Exclusion criteria

Patients admitted to the MTC in Copenhagen or Odense from other regions than Region Zealand or from other countries. Patients admitted to one of the two other MTCs in Denmark.

### Data analyses

Descriptive analyses were performed by use of numbers and percentages. Trauma patients were described by gender, age group, comorbidity, ISS-score, head trauma, penetrating trauma, and 30-day survival. The prehospital characteristics for each trauma patient were described by the dispatch code and emergency level assessed and registered by the emergency medical dispatcher during the emergency call, and the type of EMS unit and accompanying competence dispatched. The response time was calculated as the time from dispatch of the EMS unit to the arrival of the first EMS unit at the scene. The time spent at the scene was calculated from the arrival of the first EMS unit to the departure of the EMS unit transporting the patient. The time from the emergency call to arrival at the MTC was also calculated. The descriptive analysis was performed overall and stratified by primary admission or secondary transfer to an MTC. For a geographical overview, descriptive maps of the injuries or traumas were constructed, overall and for primary and secondary patients, respectively.

All data management and statistical analyses were performed in R studio v. 4.3.2.

### Ethical considerations

No formal ethical approval is needed for register-based studies, according to the regional scientific ethical committee in Region Zealand. Approval for performance of the study and storage of data was given by the Regional Research Directory in December 2023 (No. REG-115-2022).

## Results

**Fig. 1:**
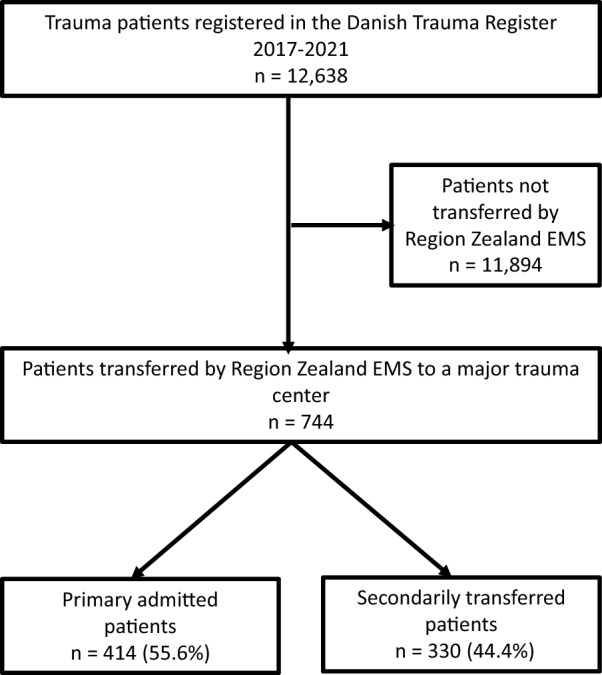
Data flowchart

Of 12,638 patients registered in the Danish Trauma Register in the study period, 744 trauma patients were encountered by the Region Zealand EMS and included in the descriptive analysis. Of these 414 (55.6%) patients were primarily admitted to an MTC, while 330 (44.4%) patients were transferred to an MTC after initial assessment at the local trauma unit (Fig. 1).

### Patient characteristics

The characteristics of “primary” and “secondary” patients are outlined in Table 1. Overall, there were more men (70.2%) than women (29.8%) among all trauma patients. Among secondary patients, there was a relatively higher proportion of women compared to primary patients (34.2% vs. 26.3%). The age distribution was similar for both groups, with approximately two-thirds of patients falling within the age range of 18–65 years. Notably, a higher percentage of secondary patients had an ISS score of ≥ 15 (59.6%) compared to primary patients (47.8%). The distribution of comorbidity scores was similar in both groups, with 13.9% and 12.6% of primary and secondary patients having mild comorbidity, and 7.3% and 9.1% of primary and secondary patients having severe comorbidity, respectively. Due to a high degree of missing data, trends for the number of penetrating trauma and head trauma were difficult to ascertain.

**Table 1:** Patient characteristics of patients primarily admitted or secondarily transferred to a major trauma center

Variable	Level	Primary admission to MTC n = 414	Secondary transfer to MTC n = 330	Total n = 744
Sex, n(%)	Female	109 (26.3)	113 (34.2)	222 (29.8)
	Male	305 (73.7)	217 (65.8)	522 (70.2)
Agegroup, n(%)	0–2	3 (0.7)	7 (2.1)	10 (1.3)
	3–12	23 (5.6)	22 (6.7)	45 (6.0)
	13–17	13 (3.1)	21 (6.4)	34 (4.6)
	18–30	90 (21.7)	48 (14.5)	138 (18.5)
	31–65	198 (47.8)	156 (47.3)	354 (47.6)
	66–75	44 (10.6)	44 (13.3)	88 (11.8)
	76 +	43 (10.4)	32 (9.7)	75 (10.1)
ISS, n (%)	< 15	128 (52.2)	78 (40.4)	206 (47.0)
	≥ 15	117 (47.8) (missing, n = 169)	115 (59.6) (missing, n = 137)	232 (53.0) (missing, n = 306)
Charlsons comorbidity score, n (%)	0 (no comorbidity)	201 (77.6)	144 (72.7)	345 (75.5)
	1 (mild comorbidity)	36 (13.9)	29 (12.6)	65 (14.2)
	≥ 2 (severe comorbidity)	22 (8.5) (missing, n = 155)	25 (14.6) (missing, n = 132)	47 (10.3) (missing, n = 287)
Head trauma, n(%)	Yes	37 (23.9)	22 (16.7)	59 (20.6)
	No	118 (76.1) (missing, n = 259)	110 (83.3) (missing, n = 198)	228 (79.4) (missing, n = 457)
Penetrating trauma, n (%)	Yes	8 (3.1)	5 (2.5)	13 (2.9)
	No	249 (96.9) (missing, n = 157)	194 (97.5) (missing, n = 131)	443 (97.1) (missing, n = 288)
30 day survival, n (%)	Yes	360 (87.0)	306 (92.7)	666 (89.5)
	No	54 (13.0)	24 (7.3)	78 (10.5)

*MTC* major trauma center, *ISS* injury severity score

Finally, we found a slightly lower proportion of patients alive after 30 days among primary patients (87.0%) compared to secondary patients (92.7%).

### Prehospital characteristics

The EMS characteristics are presented in Table 2. The most frequent dispatch codes registered for trauma patients were “Road traffic accidents” and “Accidents” in both groups, accounting for 79.4% of registered codes in total. In the group of primarily admitted patients “Psychiatry/suicide,” and “Violence/abuse” accounted for 7.2% and 4.7% of dispatch codes, respectively, whereas “Unclear problem” was registered for the secondarily admitted patient group in 6.0% of the cases vs 1.9% of the primary patients. In terms of medical dispatchers’ perception of degree of urgency, “Emergency level A” (potentially life-threatening situation) was registered in 98.1% in the group of primary patients, whereas this was the case in 86.3% of cases in the secondary patient group.

**Table 2:** Prehospital characteristics of patients primarily admitted or secondarily transferred to a major trauma center

Variable	Level	Primary admission to MTC (n = 414)	Secondary transfer to MTC (n = 330)	Total (n = 744)
Emergency level as assessed by medical dispatchers, n (%) (missing, n = 133)	Emergency level A (potentially life threatening)	356 (98.1)	214 (86.3)	570 (93.3)
	Emergency level B (urgent but not life threatening)	7 (1.9)	34 (13.7)	41 (6.7)
Danish Index Category, n (%) (missing, n = 133)	Traffic accident	154 (42.4)	91 (36.7)	245 (40.1)
	Accidents	137 (37.7)	108 (43.5)	245 (40.1)
	Psychiatry/Suicide	26 (7.2)	4 (1.6)	30 (4.9)
	Violence/Abuse	17 (4.7)	7 (2.8)	24 (3.9)
	Unclear problem	7 (1.9)	15 (6.0)	22 (3.6)
	Other categories	22 (6.1)	23 (9.4)	45 (7.4)
First competence at scene, n (%) (missing, n = 49)	MCCU	43 (10.5)	8 (2.8)	51 (7.3)
	HEMS	27 (6.6)	2 (0.7)	29 (4.2)
	APM	45 (11.0)	31 (10.9)	76 (10.9)
	PM	201 (49.0)	142 (49.8)	343 (49.4)
	Other	94 (22.9)	102 (35.8)	196 (28.2)
Highest competence at scene (missing, n = 45)	MCCU	42 (10.1)	142 (49.8)	184 (26.3)
	HEMS	286 (69.1)	34 (11.9)	320 (45.8)
	APM	13 (3.1)	51 (17.9)	64 (9.2)
	PM	41 (9.9)	49 (17.2)	90 (12.9)
	Other	32 (7.7)	9 (3.2)	41 (5.9)
Response time, first unit at scene, mm:ss, median, [Q1,Q3] (missing, n = 161)		09:22 [06:27, 13:03] (n = 339) (missing, n = 75)	08:36 [06:00, 14:00] (n = 244) (missing, n = 86)	
Time at scene, hh:mm:ss, median, [Q1,Q3] (missing, n = 147)		00:47:09 [00:28:46, 01:20:10] (n = 315) (missing, n = 99)	00:22:56 [00:15:25, 00:31:14] (n = 282) (missing, n = 48)	
Time from injury to arrival at MTC, hh:mm:ss, median [Q1,Q3] (missing, n = 45)		01:35:18 [01:17:03, 01:53:46] (n = 414) (missing, n = 0)	04:50:29 [04:03:11, 06:41:31] (n = 285) (missing, n = 45)	
Time at trauma unit, hh:mm:ss, median, [Q1,Q3]			02:52:32 [02:04:33, 04:10:50]	

*MTC* major trauma center, *HEMS* helicopter emergency medical services, *MCCU* mobile critical care unit, *APM* advanced paramedic. *PM* Paramedic

In analyzing the competencies dispatched to trauma scenes from the medical dispatch center, we found that 17.1% of primary patients were initially attended by a physician-staffed unit, (10.5% by MCCU and 6.6% by HEMS), in contrast to only 3.5% of secondary patient cases. For the highest level of competence present at the scene at any given time, physician-staffed units were present in 79.2% of cases involving primary patients, compared to 61.7% of cases involving secondary patients.

**Fig. 2:**
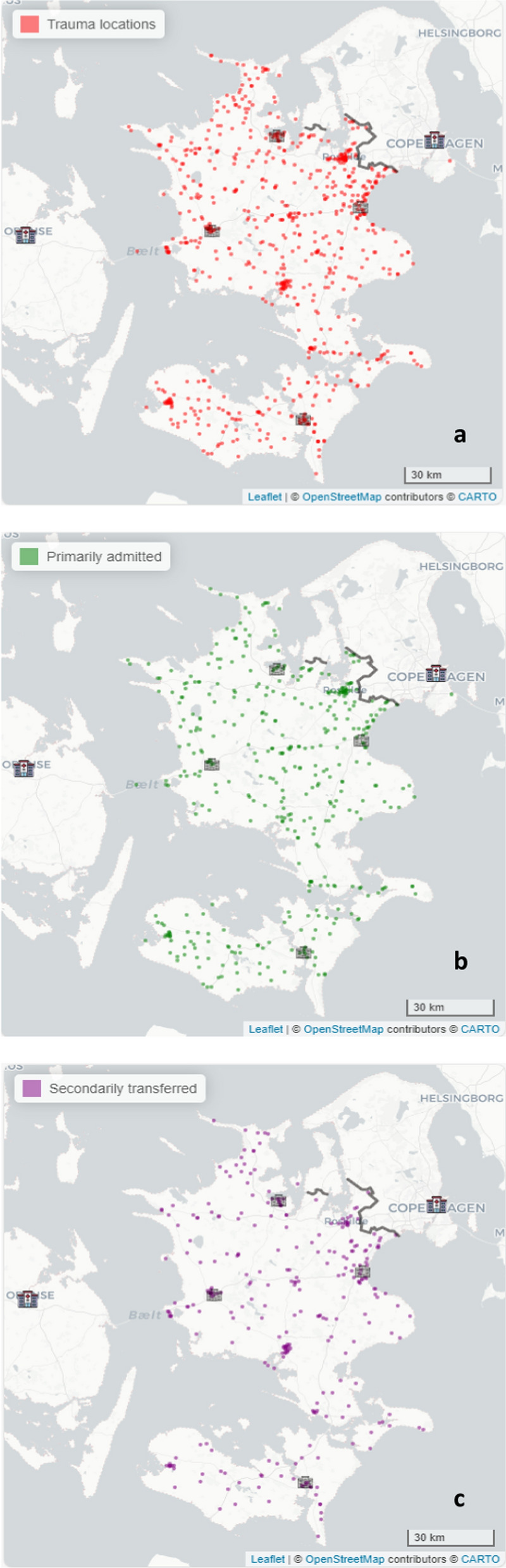
Geographical distribution of **a** all trauma **b** Primary admitted trauma patients **c** Secondarily transferred trauma patients

The median response time for the first unit at the scene was 9 m:22 s for primary patients [Q1,Q3: 6 m:27 s, 13 m:03 s] versus 8 m:36 s for secondary patients [Q1,Q3: 6 m:00 s, 14 m:00 s]. The prehospital personnel spent more time at scene with the primary patients compared to the secondary patients with a median of 47 m:09 s [Q1,Q3: 28 m:46 s, 1 h:2 m:10 s] versus 22 m:56 s [Q1,Q3: 15 m:25 s, 31 m:14 s]). Finally, we found that the median time from injury to arrival at MTC was 1 h:35 m:18 s [Q1,Q3: 1 h:17 m:03 s,1 h:53 m:46 s] for primary admitted patients, compared to 4 h:50 m:29 s [Q1,Q3: 4 h:03 m:11 s**–**6 h:41 m:31 s] for secondary patients. The geographical distribution of the trauma patients, overall and subdivided according to primary or secondary admission to the MTC are illustrated in Fig. 2.

## Discussion

This study aimed at describing severely injured patients from Region Zealand in Denmark who were either primarily admitted or secondarily transferred to an MTC. The main findings were that secondary trauma patients had a higher proportion of women, more patients with high ISS scores, and higher survival rates. Furthermore, at the time of call these cases were considered less urgent and provided with a physician-staffed EMS unit to a lesser extent compared to primary patients. Finally, secondary trauma patients had a longer time interval from the injury occurred to arrival at an MTC.

The finding of a higher ISS score among secondary trauma patients is noteworthy, given the fact that these patients were considered of lower urgency by medical dispatchers and thus provided with a lower proportion of the highest level of EMS response. However, the ISS score is a retrospective measure and perhaps the results reflect the fact that the severity of a trauma is both multifactorial and a continuum over time. The numbers are comparable with data from a recent Dutch study in which the proportion of patients with ISS > 15 was 50.5% and 33.0% for secondary and primary patients, respectively [25]. In line with our results, this study also found a higher survival rate for secondary trauma patients. The findings might emphasize the challenge in assessing a trauma patient for medical dispatchers through the emergency call and at the scene by EMS personnel without the opportunity to perform paraclinical assessment except from ultrasound and vital signs. The fact that minor injuries can cause great damage to frail elderly may be part of this challenge. Likewise, critically injured children may be difficult to recognize due to their ability to sustain major injuries without physiological decompensation. In addition, the lower frequency of pediatric trauma may play a role in the healthcare professionals' experience in identifying and treating this patient group. Another explanation could be that the ISS score was a result of damage to several body regions whose injuries by themselves do not require immediate specialized treatment, but that together they constitute a polytrauma, which in time requires transfer to an MTC. In this case, the pre-hospital triage to a trauma unit might not affect patient outcome but may be a reasonable choice in terms of transport times, rapid pain relief and stabilization of fractures or other temporary treatments. On the other hand, the fact that nearly half of the patients are potentially multi-traumatized suggests some degree of undertriage, regardless of this being during emergency calls or during EMS assessment. Both studies emphasize the need for more detailed investigation of these aspects of trauma care. The underlying factors contributing to undertriage can only be hypothesized. Suboptimal guidelines and algorithms may play a role, but the human factor may also be important, as shown in an older mixed methods study regarding pre-hospital decision-making, where it was found that provider cognitive reasoning for field trauma triage was driven primarily by provider judgment, rather than specific triage criteria [26]. At the dispatch center, improving the assessment of urgency and trauma triage could potentially be achieved by enhancing the collaboration between medical dispatchers and the on-call physician leading to better decision making. The same would apply to the corporation between ambulance personnel and the on-call physician.

Our analysis of prehospital time showed expectable results. The time at the scene was shorter for secondary patients than for primary patients, which might be explained by a decision to get the patient to the nearest hospital quickly with few prehospital interventions performed, while primary patients might be perceived to be more severely injured at scene and therefore treatment is more complex and time-consuming. The fact that more than 4 h passed from injury to arrival at the MTC for secondary patients has also been found in previous studies [16] and perhaps underlines a potential for improvement if time is associated with outcome, which is currently discussed in the medical literature. For instance a large study of torso trauma with non-compressible haemorrhage found that longer prehospital times were independently associated with higher mortality [27]. A systematic review from 2015 suggested that longer prehospital times were associated with higher mortality for patients with penetrating trauma or traumatic brain injury, whereas results were conflicting for patients with undifferentiated diagnoses, depending on which exact prehospital time measure was investigated [28].

The reason for transport to the nearest hospital with a trauma patient can be driven by many factors, but the long distance to an MTC may be part of the explanation. As seen in the geographical distribution of the trauma cases, they occur widely throughout the region, whereas the MTC can easily be over 100 km away for some of the trauma patients. For non-physician staffed vehicles, quick access to skills or resources that the paramedics do not have—e.g. airway management, anesthesia, advanced pain management, and access to blood may be a reason. Another reason why HEMS frequently transports patients to the MTC could be the faster and more convenient access offered by helicopters to the trauma center. Consequently, they might also contribute to some degree of overtriage. On the other hand, non-physician-staffed vehicles may contribute to undertriage.

## Future perspectives

To optimize trauma care in Denmark, knowledge about the entire course for trauma patients is essential, as described in the “trauma chain of survival” [29, 30]—from when the emergency medical services are alarmed, to when the patient is picked up by an ambulance, admitted and treated in a hospital, to when they are discharged for further rehabilitation. A trauma system contains all these elements, and to create an optimal trauma system for the benefit of the patient, all elements must be investigated and optimized [3, 31]. To identify the inadequacies in the system, data is required.

Knowledge about Danish trauma patients is sparse. We have information about the trauma patients who are admitted to the major trauma centers, as these patients are registered in the Danish Trauma Register. The data in the register is used to calculate predefined quality indicators for the four major trauma centers in Denmark. Unfortunately, exact data regarding trauma patients who are not admitted to an MTC are unavailable, as the registration of these is currently inadequate. By doing a broad search in prehospital data, it is possible to identify all trauma patients. However, such a search would include all cases, from broken limbs or minor wounds, to the major traumas caused by traffic accidents, and thereby the data would overestimate trauma. If searching in-hospital data for trauma patients, the number of identified patients will depend on the registration of trauma codes for each triggered trauma call by the personnel involved in trauma. Despite increasing focus on this registration, this is not yet done adequately.

To ensure high quality in trauma data, we need a consistent and thorough data registration of traumas, like seen in registers in Holland [32] and England [33]. Experience from other mature clinical registers in Denmark, such as the Danish Cardiac Arrest Register could be utilized [34]. Here, a thorough data validation is consistently carried out, the register is continuously modified, so that the registered variables are clinically relevant and well-defined. However, to maintain a register of good quality it is required to have financial support, leadership, collaboration between centers, commitment, and accountability, which might be challenging, especially for small trauma units with low patient volume and no funding.

## Limitations

This study has several limitations. First, the retrospective design implies that we cannot examine associations between variables, but only carry out descriptive analysis. Importantly, we had a lot of missing information on some variables such as the Injury Severity Score, which must also be considered in the interpretation of the results. In addition, we had to make some rough decisions in our data management, such as defining a severely injured patient as an individual who is ultimately admitted to a trauma center. We lack data regarding patients who die prior to hospital arrival, hindering survival analyses due to confounding by indication. We also lack data regarding severely injured trauma patients that are admitted to a trauma unit and never proceed to trauma center admission. Therefore, it's imperative to account for immortal time bias when interpreting the results, even in the absence of investigation of causal relationships.

Immortal time bias can lead to overestimation of the outcome event rate in the unexposed group, underestimation of the event rate in the exposed group, or both due to a period during which an individual cannot experience the event of interest, such as death [35]. Finally, incorporating stratified analyses based on injury type (hemorrhage, head injury, blunt or penetrating trauma) would have added more depth to the discussion.

## Conclusion

Secondary trauma patients had higher ISS scores and higher survival rates when compared to primary trauma patients. Furthermore, secondary trauma patients were considered less urgent and provided with a lower response type by medical dispatchers and less frequently assessed by physician-staffed units. To further investigate survival in adjusted analyses and optimal triage, prospective quality data is needed for the entire treatment course enabling continuous monitoring and optimization of quality in treatment for trauma patients.

## Data Availability

Aggregated data are available upon reasonable request.

## Appendix: Prehospital trauma triage in Region Zealand

**Table:**

Trauma team activation at Trauma Unit (prehospital triage, updated 2007)*			
Points	0	1	2
Trauma mechanism	Low energy trauma	High energy trauma^#^	
Consciousness	Awake	Unclear	Unresponsive
Breathing	Normal	Insufficient	Apnea
Circulation	Systolic blood pressure > 90 mmHg		Systolic blood pressure < 90 mmHg
Neck/cervical spine	Not sore	Pain	Paralyses/lack of sense of touch
Thorax	Not sore	Pain	Open lesion
Abdomen	Not sore	Pain	Open lesion
Extremities and pelvis	Not sore	Pain	Multiple fractures
Point, sum			
* The trauma team is activated by a sum of point of 2 or more. # Definition of high energy trauma: Fall > 4 meter Dead person in the crashed vehicle Pedestrian or cyclist hit by a car or motorcycle Entrapped patient Patient ejected from car or motorcycle Car rolled over Frontal collision against solid object Other Hourse accidents Gunshot or knive accidents Explotion accidents Increased risk (age < 6, age > 75 and comorbidity)			

Primary or secondary receival at major trauma center, Copenhagen	
Primary admission	Secondary transfer
Physiological criteria Child < 2 years or Clinical condition: Resp. frequency < 10 or > 29, or Systolic BT < 90, or GCS 3–13 Anatomical criteria Unconsciousness after relevant head trauma Cranial fractures (open or impressions) All penetrating (stab/shot) injuries to the head, neck, chest, abdomen and arms/legs above the elbow/knee Flail chest Burns (as a general rule, children > 10% and adults > 15% of BSA Suspected carbon monoxide poisoning and/or inhalation injury) Major fractures of two or more long bones Pelvic fracture (suspected) Paralysis in arms/legs after trauma Injury mechanism criteria *Traffic accidents* Person in high-speed accidents with significant damage to the vehicle (> 65 km/h or/and > 0.5 m deformation) Entrapped patient Persons ejected from the vehicle during the accident Patients in a vehicle where a driver or passenger has died Cyclist or pedestrian hit by lorry/bus/train/other vehicle Motorcycle accidents (collision or at speed > 50 km/h) *Fall from height* 2nd floor corresponding to ≥ 4 m In children: fall from ≥ three times the child's height Acts of violence Gunshot/stab wounds/explosion injuries (see also anatomical criteria) *Drowning and/or hypothermia *(≤ 32 °C) Increased attention to: Age (> 65 or < 15 years) Severe comorbidity Anticoagulant therapy Pregnancy (> 20 weeks) Concomitant intoxication (alcohol or euphoric drugs)	The major trauma center accepts patients referred from other hospitals based on the following criteria: Patients who meet the primary visitation criteria, e.g. clearly undertriaged patients or self-referrals with serious injuries who are transferred before CT scanning/surgery Multi-traumatised patients who, after a CT scan at the Trauma Unit, are diagnosed with either major lesions in at least two body regions or with major lesions in combination with burns Patients with diagnosed lesions, that require highly specialized care or observation

## Abbreviations

MTC: Major trauma center
EMS: Emergency medical service
HEMS: Helicopter emergency medical services
MCCU: Mobile critical care units

## References

[1] GBD 2016 Causes of Death Collaborators. Global, regional, and national age-sex specific mortality for 264 causes of death, 1980–2016: a systematic analysis for the Global Burden of Disease Study 2016. Lancet. 2016;390:1151–210. 10.1016/S0140-6736(17)32152-9 PMC5605883 28919116

[2] Injuries and violence [Internet]. https://www.who.int/news-room/fact-sheets/detail/injuries-and-violence. Accessed 22 Apr 2024.

[3] Choi J, Carlos G, Nassar AK, Knowlton LM, Spain DA. The impact of trauma systems on patient outcomes. Curr Probl Surg. 2021;58:100840. 33431135 10.1016/j.cpsurg.2020.100840 PMC7274082

[4] Spahn DR, Bouillon B, Cerny V, Duranteau J, Filipescu D, Hunt BJ, et al. The European guideline on management of major bleeding and coagulopathy following trauma: fifth edition. Crit Care. 2019;23:98. 30917843 10.1186/s13054-019-2347-3 PMC6436241

[5] Jeppesen E, Cuevas-Østrem M, Gram-Knutsen C, Uleberg O. Undertriage in trauma: an ignored quality indicator? Scand J Trauma Resusc Emerg Med. 2020;28:34. 32375842 10.1186/s13049-020-00729-6 PMC7204312

[6] Newgard CD, Uribe-Leitz T, Haider AH. Undertriage remains a vexing problem for even the most highly developed trauma systems: the need for innovations in field triage. JAMA Surg. 2018;153:328. 29094149 10.1001/jamasurg.2017.4499

[7] Morris RS, Karam BS, Murphy PB, Jenkins P, Milia DJ, Hemmila MR, et al. Field-triage, hospital-triage and triage-assessment: a literature review of the current phases of adult trauma triage. J Trauma Acute Care Surg. 2021;90:138–45. 10.1097/TA.0000000000003125 33605709

[8] Sorensen MJ, von Recklinghausen FM, Fulton G, Burchard KW. Secondary overtriage: the burden of unnecessary interfacility transfers in a rural trauma system. JAMA Surg. 2013;148:763–8. 23784088 10.1001/jamasurg.2013.2132

[9] Tang A, Hashmi A, Pandit V, Joseph B, Kulvatunyou N, Vercruysse G, et al. A critical analysis of secondary overtriage to a Level I trauma center. J Trauma Acute Care Surg. 2014;77:969–73. 25423540 10.1097/TA.0000000000000462

[10] Newgard CD, Fischer PE, Gestring M, Michaels HN, Jurkovich GJ, Lerner EB, et al. National guideline for the field triage of injured patients: recommendations of the national expert panel on field triage, 2021. J Trauma Acute Care Surg. 2022;93(2):e49–60. 35475939 10.1097/TA.0000000000003627 PMC9323557

[11] Voskens FJ, van Rein EAJ, van der Sluijs R, Houwert RM, Lichtveld RA, Verleisdonk EJ, et al. Accuracy of prehospital triage in selecting severely injured trauma patients. JAMA Surg. 2018;153:322–7. 29094144 10.1001/jamasurg.2017.4472 PMC5933379

[12] Garwe T, Stewart K, Stoner J, Newgard CD, Scott M, Zhang Y, et al. Out-of-hospital and inter-hospital under-triage to designated tertiary trauma centers among injured older adults: a 10-year statewide geospatial-adjusted analysis. Prehosp Emerg Care. 2017;21:734–43. 28661712 10.1080/10903127.2017.1332123 PMC5668189

[13] van der Sluijs R, van Rein EAJ, Wijnand JGJ, Leenen LPH, van Heijl M. Accuracy of pediatric trauma field triage: a systematic review. JAMA Surg. 2018;153:671–6. 29799916 10.1001/jamasurg.2018.1050

[14] Meisler R, Thomsen AB, Abildstrøm H, Guldstad N, Borge P, Rasmussen SW, et al. Triage and mortality in 2875 consecutive trauma patients. Acta Anaesthesiol Scand. 2010;54:218–23. 19817720 10.1111/j.1399-6576.2009.02075.x

[15] Arleth T, Rudolph SS, Svane C, Rasmussen LS. Time from injury to arrival at the trauma centre in patients undergoing interhospital transfer. Dan Med J. 2020;67:A03200138. 32862836

[16] The Strengthening the Reporting of Observational Studies in Epidemiology (STROBE) Statement. https://www.equator-network.org/. Accessed 30 June 2024.

[17] Andersen MS, Johnsen SP, Sørensen JN, Jepsen SB, Hansen JB, Christensen EF. Implementing a nationwide criteria-based emergency medical dispatch system: a register-based follow-up study. Scand J Trauma Resusc Emerg Med. 2013;21:53. 23835246 10.1186/1757-7241-21-53 PMC3708811

[18] Møller TP, Ersbøll AK, Tolstrup JS, Østergaard D, Viereck S, Overton J, et al. Why and when citizens call for emergency help: an observational study of 211,193 medical emergency calls. Scand J Trauma Resusc Emerg Med. 2015;23:88. 26530307 10.1186/s13049-015-0169-0 PMC4632270

[19] Visitationskriterier og Traumemanual [Internet]. https://www.rigshospitalet.dk/afdelinger-og-klinikker/hovedorto/traumecenter-og-akut-modtagelse/for-fagfolk/Sider/primaere-visitationskriterier.aspx. Accessed 22 Apr 2024.

[20] Dansk Traumeregister (DTR) [Internet]. https://www.rkkp.dk/kvalitetsdatabaser/databaser/dansk-traumeregister/. Accessed 22 Apr 2024.

[21] Baker SP, O’Neill B, Haddon W, Long WB. The injury severity score: a method for describing patients with multiple injuries and evaluating emergency care. J Trauma. 1974;14:187–96. 4814394 10.1097/00005373-197403000-00001

[22] Charlson ME, Pompei P, Ales KL, MacKenzie CR. A new method of classifying prognostic comorbidity in longitudinal studies: development and validation. J Chronic Dis. 1987;40:373–83. 3558716 10.1016/0021-9681(87)90171-8

[23] Lindskou TA, Mikkelsen S, Christensen EF, Hansen PA, Jørgensen G, Hendriksen OM, et al. The Danish prehospital emergency healthcare system and research possibilities. Scand J Trauma Resusc Emerg Med. 2019;27:100. 31684982 10.1186/s13049-019-0676-5 PMC6829955

[24] Pedersen CB. The Danish civil registration system. Scand J Public Health. 2011;39:22–5. 21775345 10.1177/1403494810387965

[25] van den Driessche CRL, Sewalt CA, van Ditshuizen JC, Stocker L, Verhofstad MHJ, Van Lieshout EMM, et al. Primary admission and secondary transfer of trauma patients to Dutch level I and level II trauma centers: predictors and outcomes. Eur J Trauma Emerg Surg Off Publ Eur Trauma Soc. 2022;48:2459–67. 10.1007/s00068-021-01790-1 PMC9192481 34586442

[26] Newgard CD, Nelson MJ, Kampp M, Saha S, Zive D, Schmidt T, et al. Out-of-hospital decision making and factors influencing the regional distribution of injured patients in a trauma system. J Trauma. 2011;70:1345–53. 21817971 10.1097/TA.0b013e3182191a1b PMC3151488

[27] Alarhayem AQ, Myers JG, Dent D, Liao L, Muir M, Mueller D, et al. Time is the enemy: Mortality in trauma patients with hemorrhage from torso injury occurs long before the “golden hour.” Am J Surg. 2016;212:1101–5. 27832843 10.1016/j.amjsurg.2016.08.018

[28] Harmsen AMK, Giannakopoulos GF, Moerbeek PR, Jansma EP, Bonjer HJ, Bloemers FW. The influence of prehospital time on trauma patients outcome: a systematic review. Injury. 2015;46:602–9. 25627482 10.1016/j.injury.2015.01.008

[29] Bakke HK, Wisborg T. We need to include bystander first aid in trauma research. Scand J Trauma Resusc Emerg Med. 2017;25:32. 28335785 10.1186/s13049-017-0372-2 PMC5364713

[30] Latif RK, Clifford SP, Baker JA, Lenhardt R, Haq MZ, Huang J, et al. Traumatic hemorrhage and chain of survival. Scand J Trauma Resusc Emerg Med. 2023;31:25. 37226264 10.1186/s13049-023-01088-8 PMC10207757

[31] Dijkink S, Nederpelt CJ, Krijnen P, Velmahos GC, Schipper IB. Trauma systems around the world: a systematic overview. J Trauma Acute Care Surg. 2017;83:917–25. 28715361 10.1097/TA.0000000000001633

[32] Driessen MLS, Sturms LM, Bloemers FW, Ten Duis HJ, Edwards MJR, den Hartog D, et al. The Dutch nationwide trauma registry: the value of capturing all acute trauma admissions. Injury. 2020;51:2553–9. 32792157 10.1016/j.injury.2020.08.013

[33] Mullen S, Tolson A, Bouamra O, Watson B, Lyttle MD, Roland D, James D. Comparison of injury patterns and interventions between adolescent, adult and paediatric trauma cases: a cross-sectional review of TARN data. BMJ Open. 2023;13:e064101. 37160391 10.1136/bmjopen-2022-064101 PMC10173954

[34] Jensen TW, Blomberg SN, Folke F, Mikkelsen S, Rostgaard-Knudsen M, Juelsgaard P, et al. The national Danish cardiac arrest registry for out-of-hospital cardiac arrest: a registry in transformation. Clin Epidemiol. 2022;14:949–57. 35966902 10.2147/CLEP.S374788 PMC9374329

[35] Yadav K, Lewis RJ. Immortal time bias in observational studies. JAMA. 2021;16:686–7. 10.1001/jama.2020.9151 33591334

